# NPC2 as a Prognostic Biomarker for Glioblastoma Based on Integrated Bioinformatics Analysis and Cytological Experiments

**DOI:** 10.3389/fgene.2021.611442

**Published:** 2021-03-11

**Authors:** De Wei, Shanghang Shen, Kun Lin, Feng Lu, Pengfeng Zheng, Shizhong Wu, Dezhi Kang

**Affiliations:** ^1^Department of Neurosurgery, Fujian Provincial Hospital South Branch, Fuzhou, China; ^2^Department of Neurosurgery, Shengli Clinical Medical College of Fujian Medical University, Fuzhou, China; ^3^Department of Neurosurgery, The First Affiliated Hospital of Fujian Medical University, Fuzhou, China; ^4^Department of Neurosurgery, Shanghai General Hospital, Shanghai Jiao Tong University School of Medicine, Shanghai, China

**Keywords:** glioblastoma, prognostic biomarkers, NPC2, Gene Expression Omnibus (GEO), bioinformatics analysis

## Abstract

Glioblastoma (GBM) is one of the most common and fatal malignancies worldwide, while its prognostic biomarkers are still being explored. This study aims to identify potential genes with clinical and prognostic significance by integrating bioinformatics analysis and investigating their function in HNSCC. Based on the Single-cell RNA sequencing (scRNA-seq) results of H3K27M-glioma cells, computational bioinformatics methods were employed for selecting prognostic biomarker for GBM. The protein NPC2 (NPC Intracellular Cholesterol Transporter 2), which has been shown to be related to lipoprotein metabolism and innate immune system, was identified to be upregulated in GBM. NPC2 showed a relatively higher expression in GBM samples, and a negative correlation with tumor purity and tumor infiltrating immune cells. Additionally, NPC2 was knocked down in U87-MG and U251 cells line, and cell proliferation and migration capability were evaluated with CCK-8, scratch and transwell assay, respectively. Cytological experiments has shown that NPC2 overexpression inhibited GBM cells proliferation and migration, indicating its important role in GBM progression. This is the first investigation into the prognostic value of NPC2 interact with GBM. The potential molecular factor NPC2 have been identified as a prognostic biomarker for GBM.

## Introduction

Glioblastoma (GBM) is the most common and lethal primary brain malignancy with standard therapeutic regimen including surgical resection, followed by radiotherapy, with concurrent chemotherapy (temozolomide) and maintenance therapy (temozolomide for 6−12 months) (Abdul et al., 2018; Santangelo et al., 2020). Despite an aggressive multimodal standard therapy, patients with GBM have a dramatically poor prognosis with a median overall survival of only 13−15 months after standard therapy (Geraldo et al., 2019; Albright et al., 2020). The complicated genetic and molecular changes within cancer cells lead to the complex biology of GBM. The Cancer Genome Atlas (TCGA) initiative has provided a higher-resolution picture of driven characterization of rapid advancements in GBM biomarker discovery with distinct expression signatures (Chen Z. et al., 2020). However, the prognosis for GBM patients is still poor. Hence, the discovery of more sensitive prognostic biomarkers for GBM remains crucial. Currently, age, metastasis, and tumor resection have been identified as prognostic factors for GBM (Lakomy et al., 2020; Li et al., 2020; Shen et al., 2020). What’s more, genetic researches have also promoted the disclosure of a few sporadic biomarkers for GBM (Mirchia and Richardson, 2020; Zhao et al., 2020).

The protein NPC2 (NPC Intracellular Cholesterol Transporter 2) is a protein coding gene containing a lipid recognition domain, which has been shown to be related to lipoprotein metabolism and innate immune system pathways. NPC2 plays an important role in regulating the transport of cholesterol through the late endosomal/lysosomal system. Mutations in this gene have been associated with Niemann-Pick disease, type C2, and frontal lobe atrophy (Infante et al., 2008; Gong et al., 2016; Li et al., 2016). In the past decades, more and more researchers have focused on the biological genetic functions of genes involved in the tumorigenesis of GBM with the development of high-throughput sequencing (Diehn et al., 2000; Diboun et al., 2006; Couturier et al., 2020). However, the link between NPC2 and prognosis of GBM has not been reported yet. This is the first investigation into the prognostic value of NPC2 interact with GBM.

The aim of this study is to explore the prognostic and carcinogenic potential of robust molecular signature that can improve the ability to predict GBM prognosis. We downloaded the single-cell RNA sequencing in 3,321 cells from six primary H3K27M-glioma and matched models profiling datasets about GBM tumor and normal tissues from the Gene Expression Omnibus (GEO). Eight overlapping differentially expressed genes between tumor and non-tumor tissues were explored, these novel potential prognostic markers were identified via multiple bioinformatics analysis, including biological process functional annotation and pathway enrichment analysis, as well as gene expression profiling interactive survival analysis. We have investigated the association between gene NPC2 expression and GBM tumorigenesis, and the prognosis role of NPC2 by integrating the GBM samples from the GEO dataset with Single-cell RNA sequencing (scRNA-seq) results of H3K27M-glioma cells. Differential expression, correlation with tumor infiltrates, prognostic significance, cell proliferation, and migration capability were evaluated with CCK-8, scratch wound healing assay, and transwell assay.

## Materials and Methods

### Data Preprocessing and Screening Differentially Expressed Genes

We downloaded the gene expression profiles of single-cell RNA sequencing in 3,321 cells from six primary H3K27M-glioma and matched models from the Gene Expression Omnibus (GEO)^1^ with accession number of GSE102130 (Filbin et al., 2018). GSE102130 dataset was composed of single-cell RNA sequencing from 2,458 single cells (K27M glioma patients) and from several models derived from BCH869—including patient derived xenograft (PDX), gliomaspheres (GS), and differentiated glioma cells (DGC). The differentially expressed genes (DEGs) were obtained by comparing the malignant cells with the non-malignant cells based on threshold of *p*-value <0.05 and absolute value of fold change >2 (Supplementary Table 1).

### Comprehensive Analysis

GEPIA is a web server and an online database^2^. GEPIA adopts a standard processing pipeline to analyze the RNA-Seq expression data from GTEx and TCGA, which included 8,587 normal and 9,736 tumor samples (Subramanian et al., 2005). We obtained samples from TCGA and used GEPIA to analyze the connections between Overall Survival (OS) and Disease Free Survival (DFS) with NPC2 expression in GBM. Additionally, a boxplot using disease state as a variable was graphed to calculate differential expression of NPC2. A *p*-value <0.05 was considered statistically significant. Our study was in accordance with the publication guidelines provided by TCGA.

### Correlation Analysis Between NPC2 and Immune Infiltration

TIMER database was used to detect the tumor immune infiltration using mRNA sequencing (mRNA-Seq) data from TCGA. TIMER applies deconvolution statistical methods previously published to infer the abundance of tumor infiltrating immune cells (TIICs) from gene expression profiles.

### Cell Culturing

Human GBM cell lines U87-MG and U251 were purchased from the Type Culture Collection of the Chinese Academy of Sciences (Shanghai, China). Cell lines were cultured in minimum essential medium-alpha with 10% heat-inactivated fetal bovine serum, 100 IU/mL penicillin, and 100 μg/mL streptomycin. Humidified incubators were supplied with 95% air atmosphere and 5% carbon dioxide at 37°C.

### Cell Transfection

U87-MG and U251 cells at exponential stage were used for transfection. Before transfection, 1 × 10^6^ cells were cultured in 6−well plates with 2 mL complete medium for 24 h until they were 90% confluent. Lentivirus-mediated NPC2 (sh-NPC) and non-targeting shRNA (sh-NC) were obtained from GenePharma (Shanghai, China).

### Cell Counting Kit-8 (CCK-8) Assay

Cellular viability was determined by the Cell Counting Kit-8 (Beyotime Biotechnology). After transfection, 100 μL of U87-MG or U251 cells per well were plated into 96-well plates at a density of 500 cells per well. 10 μL of CCK8 solution was incubated with cell medium daily to each well for another 2 h at 37°C. The absorbance of each well was detected at 570 nm by the Multiskan FC Microplate spectrophotometer. Data were presented as mean ± SD and comparisons were calculated by two-way RM ANOVA by the GraphPad Prism.

### Scratch Wound Healing Assay

U251 and U87-MG cells were evenly planted in a 6-well plate with 1 × 10^6^ cells per well. The plate was vertically scratched with a 10 μL sterile pipette tip when the cells covered 90% of the plate bottom area. After that, the culture medium in the plate was discarded and gently washed with PBS for three times, and the cell debris residue was rinsed off to make sure the visual field clear during photographing. The culture medium containing 1% FBS was added to the six-well plate. A 3 mm wound was introduced across the diameter of each plate. Cell migration was observed by microscopy at 24 h.

### Transwell Assay

Cells in logarithmic growth phase were seeded at the upper transwell chamber insert (Corning, United States) at a density of 2 × 10^4^ cells per well. The chamber was placed in a 24-well plate in which the upper chamber contained serum-free cell culture medium and the lower chamber contained 10% FBS complete medium. The culture was continued for 24 h. The medium was discarded, and stained with a crystal violet solution to observe the number of migrated cells.

### Statistical Analysis

Data were presented as mean ± SD and comparisons were calculated by Student’s *t* test (two sided, unpaired) or two-way RM ANOVA by the GraphPad Prism. Using multivariate Cox analysis, we evaluated NPC2 expression along with other pathological and clinical factors affected OS. A *p*-value <0.05 for NPC2 expression was set as the threshold. The correlations between clinical characteristics and NPC2 expression were analyzed using logistic regression.

## Results

### Overall Characteristics of the Cell Cluster by Reanalysis

**FIGURE 1:**
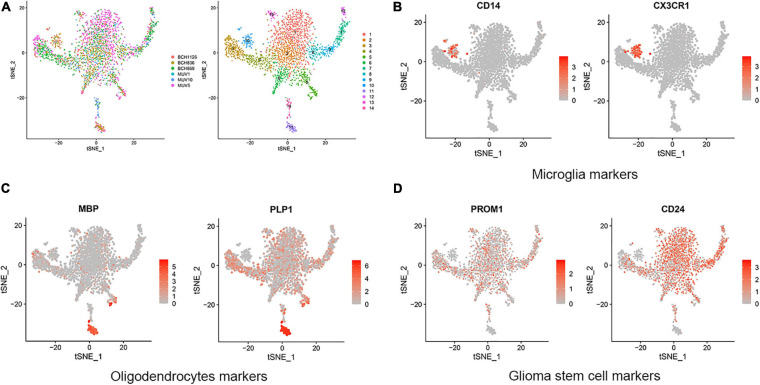
Cell clusters and cell types distribution in six GBM tissue sample. **(A)** A total of 14 cell clusters was identified and shown with t-SNE maps in six GBM tissue samples. **(B–D)** T-SNE map showing canonical markers for three cell types: microglia **(B)**, oligodendrocytes **(C)**, and glioma stem cells **(D)**.

In this research, GBM cortex of single cell RNA sequencing was sampled from 3,321 cells from six primary H3K27M-glioma and normal models in the GSE102130 dataset. Analyzed by using the Gene Expression Omnibus (GEO) database^3^, 14 cell clusters (clusters 1−14) were characterized. According to canonical cell biomarkers such as CD14 (Mathews et al., 2019), CX3CR1 (Murai et al., 2020), MBP (García-León et al., 2020), PLP1 (Guo et al., 2020), PROM1 (Bao et al., 2006), CD24 (Tiburcio et al., 2020), three tumor cell types were identified by unsupervised clustering and expression of lineage-specific markers following batch correction in 14 clusters, including microglia, oligodendrocytes, and glioma stem cells (Figure 1).

### Common DEGs in a Single-Cell RNA Sequencing Reanalysis and TCGA Database of GBM

**FIGURE 2:**
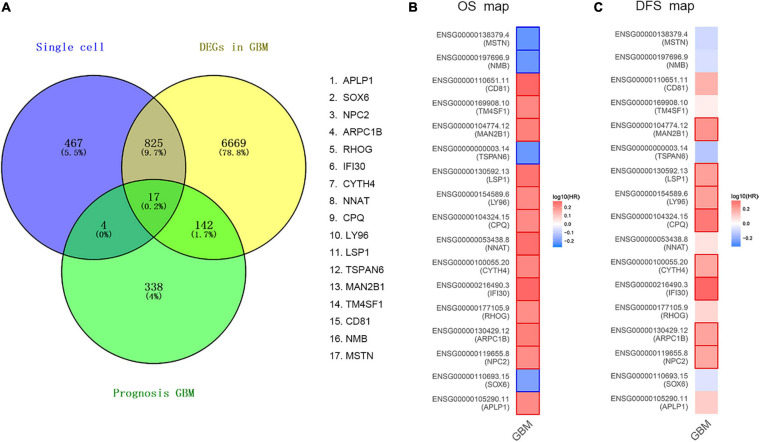
Validation of common DEGs. **(A)** The common genes between our DEGs, TCGA cohort DEGs, and PRGs of GBM. Different color areas represent different datasets. The cross areas showed the commonly changed DEGs. DEGs were identified with classical *t* test; statistically significant DEGs were defined with *P* < 0.001 and | log2 fold change| > 1 as the cut-off criteria. **(B)** Overall Survival (OS) map of common DEGs. **(C)** Disease Free Survival (DFS) map of common DEGs.

In order to determine the DEGs most highly associated with the prognosis GBM, we reanalyzed single-cell RNA sequencing based on our DEGs, TCGA cohort DEGs, and PRGs of GBM. As a result of which, 17 common DEGs were characterized, as shown in Figure 2A, including APLP1, SOX6, NPC2, ARPC1B, RHOG, IFI30, CYTH4, NNAT, CPQ, LY96, LSP1, TSPAN6, MAN2B1, TM4SF1, CD81, NMB, and MSTN. Then, we compared the OS and DFS maps of the 17 common DEGs and found that the expression trends of eight common DEGs were consistent totally. As shown in Figures 2B,C, including MAN2B1, LSP1, LY96, CPQ, CYTH4, IFI30, ARPC1B, and NPC2.

### DEGs Identification

**FIGURE 3:**
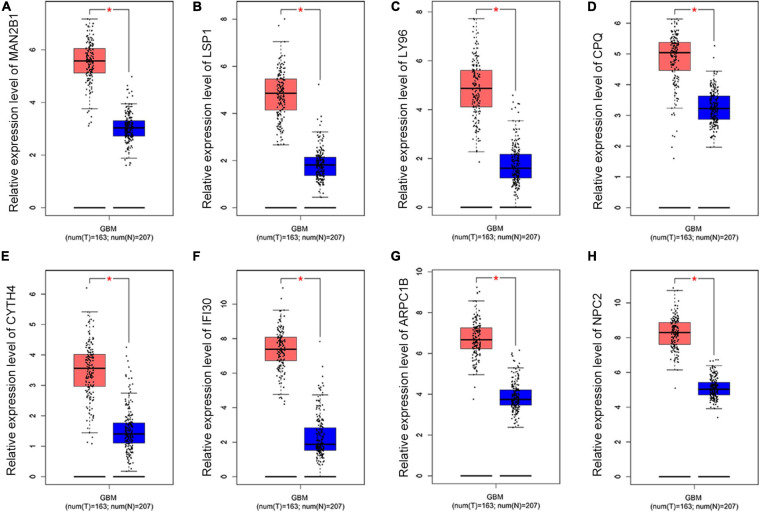
The relative expression level of eight common DEGs. Boxplots illustrating the relative expression level of **(A)** MAN2B1; **(B)** LSP1; **(C)** LY96; **(D)** CPQ; **(E)** CYTH4; **(F)** IFI30; **(G)** ARPC1B; **(H)** NPC2 in normal and GBM tissues based on the GEPIA database. *Significant difference, *p* < 0.05.

**FIGURE 4:**
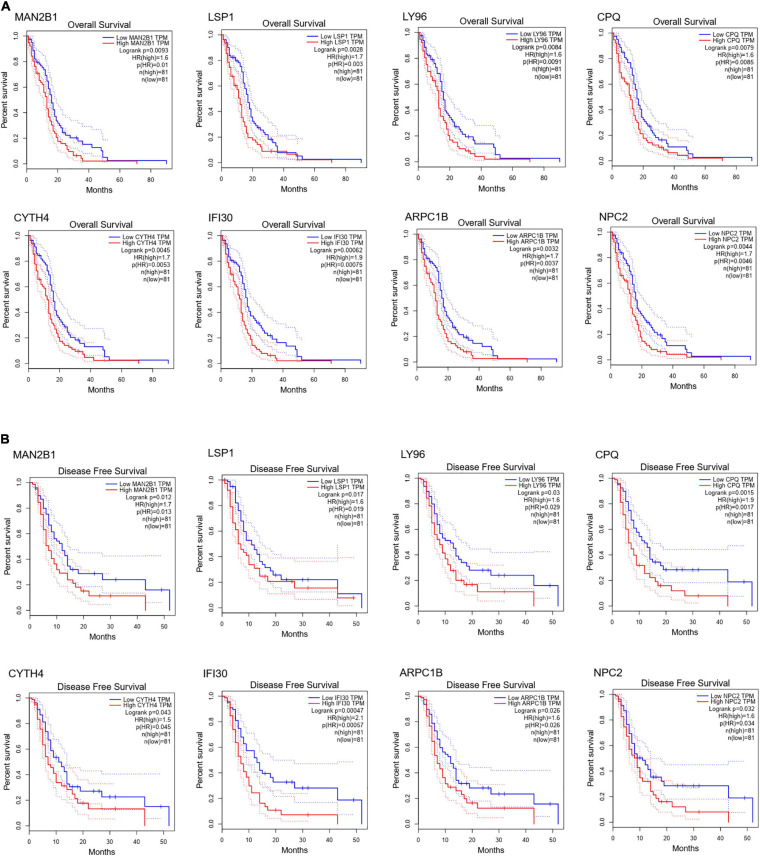
Survival curves of OS **(A)** and DFS **(B)** of eight common DEGs. Survival curves of OS **(A)** and DFS **(B)** of MAN2B1; LSP1; LY96; CPQ; CYTH4; IFI30; ARPC1B; NPC2 in normal and GBM tissues based on TCGA data in GEPIA. Red line indicates the samples with gene highly expressed, and blue line shows the samples with gene lowly expressed. HR, hazard ratio.

To further dig out the roles of these common DEGs in GBM, we analyzed the relative expression level expression level of the eight common DEGs in normal and GBM tissues based on GEPIA database. We found out that the eight common DEGs mRNA expression were also significantly increased in GBM when compared to the normal group using the GEPIA database (*p*-value <0.01, |Log2FC| > 1) (Figure 3). What’s more, a strong correlation exists between their mRNA levels and OS or DFS (*p*-value <0.001, Figure 4), including MAN2B1(OS, HR = 1.6, LogRank P = 0.0093; DFS, HR = 1.7,LogRank P = 0.012), LSP1(OS, HR = 1.7, LogRank P = 0.0028; DFS, HR = 1.6, LogRank P = 0.017), LY96(OS, HR = 1.7, LogRank P = 0.0028; DFS, HR = 1.6, LogRank P = 0.03), CPQ(OS, HR = 1.6, LogRank P = 0.0079; DFS, HR = 1.9, LogRank P = 0.0017), CYTH4(OS, HR = 1.7, LogRank P = 0.0045; DFS, HR = 1.5, LogRank P = 0.0043), IFI30(OS, HR = 1.9, LogRank P = 0.00062; DFS, HR = 2.1, LogRank P = 0.00057), ARPC1B(OS, HR = 1.7, LogRank P = 0.0032; DFS, HR = 1.6, LogRank P = 0.026), and NPC2(OS, HR = 1.7, LogRank P = 0.0046; DFS, HR = 1.6, LogRank P = 0.0032). GBM patients with a higher expression of these eight common DEGs showed a shorter survival rate, compared with patients with a lower gene expression, suggesting these eight genes as hazardous prognosticator in GBM patients.

### Tumor Infiltrates Correlation of NPC2 Expression

**FIGURE 5:**
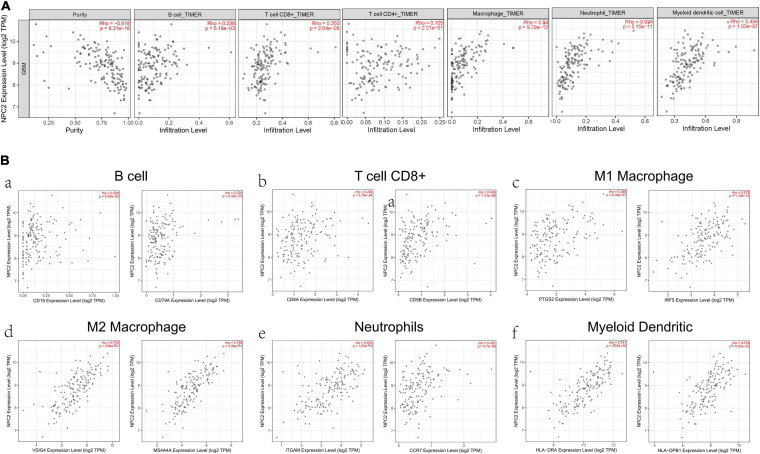
Correlation between immune cell and NPC2 expression. **(A)** The relationship between different functional immune cells, such as B cell, T cell CD8+, T cell CD4+, Macrophage, Neutrophil, and Myeloid dendritic cell with NPC2 expression in GBM. **(B)** The correlation NPC2 expression with canonical markers for six cell types: B cell, T cell CD8+, M1 Macrophage, M2 Macrophage Neutrophil, and Myeloid dendritic cell.

After searching in Pubmed, we found that the expression and function of LSP1 (Park et al., 2014), LY96 (Rajaraman et al., 2009), IFI30 (Liu et al., 2020; Zhu et al., 2020), and ARPC1B (Lauber et al., 2018) in GBM has been reported, and research about NPC2 in GBM are remain rescue. Hence, we focus on investing the clinical prognostic significance of the NPC2 genes in GBM. As shown in Table 1, by analyzing the NPC2 expression level and clinicopathological factors in 160 GBM patients, we found that NPC2 was correlated with patents’ age (*p* value = 0.04) and alive status (*p* value = 0.039). Additionally, considering the HR in OS and DFS maps were both higher than 1, we decided to choose NPC2 as the object to study in the following research. Then, we analyzed the correlation of NPC2 expression with tumor purity and tumor infiltrating immune cells. As expected, significant positive correlation of NPC2 expression with tumor purity was shown. What’s more, the correlation among NPC2 expression and tumor infiltrating B cell, CD8 + T cell, macrophages, neutrophils, and dendritic cells in GBM cancer samples was also obvious (Figure 5A). Furthermore, as the significant correlation in tumor infiltrating cell except CD4 + cell, correlation of B cell, T cell CD8+, M1 and M2 macrophages, neutrophil cells, and dendritic cells markers with NPC2 expression were analyzed. As exhibited in Figure 5B, the markers of B cell (CD19, CD79A), CD8 + T cell(CD8A, CD8B), M1 macrophages (PTGS2, IRF5), M2 macrophages (VSIG4, MS4A4A), neutrophil cells (ITGAM, CCR7), and myeloid dendritic cells (HLA-DPB1, HLA-DRA) all showed a significant positive correlation coefficient with NPC2, implicating potential role of NPC2 in tumor infiltration.

**TABLE 1:** NPC2 expression level and clinicopathological factors in 160 GBM patients.

**Characteristics**	**Case (No.)**	**NPC2 expression level**		***p* value**
		**Low (No. cases)**	**High (No. cases)**	
Age				0.04
<60	77	45	32	
60	83	35	48	
Gender				0.749
Female	54	25	29	
Male	100	49	51	
Status				0.039
Alive	31	20	11	
Dead	121	53	68	
Longest dimension				0.191
Low	42	19	23	
High	96	55	41	

### NPC2 Inhibits GBM Cancer Cell Proliferation, Migration, and Invasion

**FIGURE 6:**
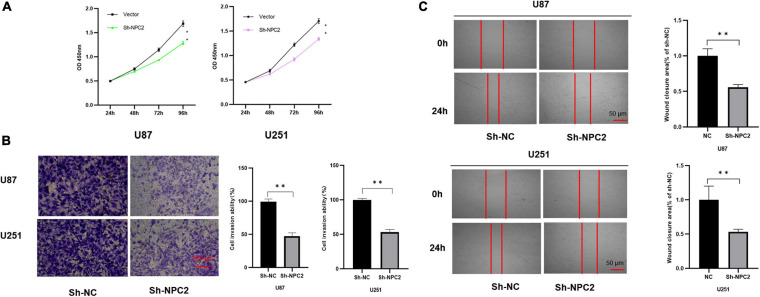
NPC2 inhibits U87-MG and U251 cells proliferation, migration, and invasion *in vitro*. **(A)** The effect of NPC2 knockdown on cell growth of U87-MG and U251cells detected by the CCK-8 assay. **(B)** The invasive and migration capacities were detected in U87-MG and U251 cells transfected with sh-NPC2 using transwell assays. **(C)** Silencing NPC2 attenuated wound closure corroborated in U87-MG and U251. The error bars indicate the mean ± SD (*n* = 3), and each experiment was repeated at least three times. *p < 0.05, **p < 0.01.

In order to verify the function of NPC2 in GBM, we silenced NPC2 in GBM cells. U87-MG and U251 are cell lines often used in the study of GBM (Gao et al., 2020; Tu et al., 2020; Xia et al., 2020; Zhang G. et al., 2020). CCK-8 assay has shown that cell proliferation was inhibited in U87-MG and U251 cells with Sh-NPC2 transfection compared with that in negative control (Sh-NC) (Figure 6A). Consistently, we performed the transwell migration assays to explore the potential effect of NPC2 on the on migration and invasion in U87-MG and U251 cells. As seen in Figure 6B, in transwell assays, the number of invading cells in the Sh-NPC2 group was less than that in the Sh-NC group. Similarly, wound healing assay showed that the metastasized cells were significantly reduced with about half of the wound closure potential when knockdown of NPC2 (Figure 6C). Together, the above results indicated that knockdown of NPC2 might inhibit growth, invasion, and migration abilities of U87-MG and U251 cells in GBM.

## Discussion

GBM is one of the most commonly seen primary malignant brain tumor in adults with an overall poor prognosis (Ostrom et al., 2018; Zhang et al., 2019; Jin et al., 2020). The recurrence rate of GBM is particularly high. Almost all GBMs recur within the first year following diagnosis and second surgical resection (Ito et al., 2019). Accurate and effective prognosis assessment play an important role in individualized treatment and follow-up treatment of patients with GBM. GBM is usually diagnosed in the late stage by MRI with poor prognosis, therefore, a reliable prognostic marker is essential (Jiang et al., 2020). Indeed, effective prognostic care for GBM patients requires a better understanding of the molecular mechanism of GBM. With the rapid development of next generation sequencing and other “omics” profiling methods, we began to focus on single-cell RNA sequencing and genomic analysis of GBM cells to investigate the detailed or new causes of GBM pathogenesis. Genetic researches have also promoted the disclosure of a few sporadic biomarkers for GBM (Cao et al., 2020b). Trying to develop new prognostic markers for prognosis treatments of this cancer is necessary.

In this research, integrated bioinformatics analysis was performed to investigate the potential gene biomarker based on single-cell RNA sequencing dataset GSE102130, TCGA cohort DEGs, and PRGs of GBM. As a result, 14 cell clusters were identified in six GBM tissue samples and we found 17 common DEGs (Figures 1, 2A). Eight DEGs were identified to be the potential protective prognostic marker of GBM while patients with a higher expression of these eight common DEGs showed shorter survival rate (Figures 3, 4). After searching in Pubmed, we found out that the expression and function of LSP1 (Park et al., 2014), LY96 (Rajaraman et al., 2009), IFI30 (Liu et al., 2020; Zhu et al., 2020), and ARPC1B (Lauber et al., 2018) in GBM has been reported, while research about NPC2 in GBM remain rescue. Gao et al. identified LSP1 as an independent predictive factor for progressive malignancy in glioma (Cao et al., 2020a). Liu et al. found the expression of IFI30 to be high in glioblastomas and in gliomas with a mesenchymal subtype or wild-type isocitrate dehydrogenase, all of which indicated the malignancy and poor outcomes of glioma (Liu et al., 2020). Tumor infiltrating immune cells in tumor microenvironment were reported to be involved in GBM progression and prognosis prediction (Fu et al., 2020; Shu and Li, 2020; Zhang Y. et al., 2020). Immune cells such as microglia, peripheral macrophages, leukocytes, and myeloid-derived suppressor cells play an important role in infiltrating gliomas. They create an immunosuppressive microenvironment to facilitate GBM cell growth and invasion and were used for designing novel immunotherapeutic approaches (Domingues et al., 2016; Gieryng et al., 2017; Quail and Joyce, 2017). NPC2 is a protein coding gene which has been shown to be related to lipoprotein metabolism and innate immune system pathways. It plays a significant role in regulating the transport of cholesterol through the late endosomal/lysosomal system in the past researches (Lauber et al., 2018; Liu et al., 2020). Its predicting value of prognosis and treatment response in glioblastoma as a novel immune-related target was also verified (Zhu et al., 2020).

In this research, we found that NPC2 was negatively correlated with tumor purity and then were functionally investigated in tumor infiltrating immune cells (Figure 5). Our result also showed that NPC2 independently indicated unfavorable prognosis in glioma when adjusted for with patents’ age (*p* value = 0.04) and alive status (*p* value = 0.039) (Table 1), which revealed that NPC2 was a robust independent factor for predicting GBM survival. Considering that age has been identified as prognostic factors for GBM (Chen P. et al., 2020; Hao et al., 2020; Zheng et al., 2020), NPC2 might be a prognostic marker of GBM based on its significant positive correlation with this disease. Hence, we focus on investing the clinical prognostic significance of the NPC2 genes in GBM.

In the next step, we verified NPC2 as a prognostic biomarker in GBM through cytological experiments, which enabled us to determine its clinical value in GBM. As the results shown, cytological experiments including CCK-8 assay, transwell assay, and scratch healing illustrated that the knockdown of NPC2 might inhibit growth, invasion, proliferation, and migration abilities of U87-MG and U251 cells in GBM, supporting it as a prognostic biomarker for GBM. Currently, we have known a little about the role of NPC2 in GBM. It was the first time that reported NPC2 as a prognostic biomarker for GBM.

In conclusion, based on bioinformatics analysis and cytological experiments, we identified NPC2 as a novel biomarker with prognostic significance in GBM.

## Data Availability Statement

The original contributions presented in the study are included in the article/Supplementary Material, further inquiries can be directed to the corresponding author/s.

## Author Contributions

DW, KL, and DK designed the research. SS, KL, and FL performed the experiments. FL, PZ, and SW visualized the results. DW, SS, and DK wrote the manuscript. DK and SS provided experimental resource. All authors reviewed and approved the final manuscript.

## Conflict of Interest

The authors declare that the research was conducted in the absence of any commercial or financial relationships that could be construed as a potential conflict of interest.

## Footnotes

1 www.ncbi.nlm.nih.gov/geo/

2 http://gepia.cancerpku.cn/

3 https://www.ncbi.nlm.nih.gov/gds
